# Supplementary material for: Determinants and Dynamic Changes of Generic Quality of Life in Human Bladder Cancer Patients
Source: J Clin Med. 2021 Nov 23;10(23):5472. doi: 10.3390/jcm10235472 (PMC8658139; doi:10.3390/jcm10235472)
Supplement: Supplementary file 1 [file jcm-10-05472-s001.zip › jcm-1437448-supplementary.pdf]

Table S1. Comparison of demographics characteristics of bladder cancer patients according to number of interviews

Parameters	All patients			NMIBC			MIBC or more		
	single	> 1	*p value*	single	> 1	*p value*	single	> 1	*p value*
Total no. patients	81	262		55	179		26	83	
Total no. measurements	81	1104		55	733		26	371	
Age (year); Mean ± SD	68.9±11.7	66.5±11.3	0.11	67.2±11.7	66.9±11.7	0.48	72.4±11.1	68.0±10.4	0.06
Age group			0.30			0.54			0.03
≥70 y/o	40	104		22 (40.0%)	69 (38.5%)		18 (69.3%)	35 (42.2%)	
60-69 y/o	21	78		18 (32.7%)	48 (26.8%)		3 (11.5%)	30 (36.1%)	
<60 y/o	20	80		15 (27.3%)	62 (34.6%)		5 (19.2%)	18 (21.7%)	
Gender (male/female)	58/23	180/82	0.62	42/13	117/62	0.13	16/10	63/20	0.15
Education (year)	8.5±5.2	9.2±4.6	0.21	9.5±5.0	9.5±4.6	0.96	6.2±5.1	8.5±4.7	0.04
Marital status (Married or cohabited/ others)	60/21	190/72	0.78	44/11	50/129	0.24	16/10	61/22	0.24
Monthly family income>NTD $50000 (yes/ no/ missing)	24/55/2	82/174/6	0.78	20/35/0	63/112/4	0.96	4/20/2	19/62/2	0.48
Vital status (dead)	23 (28.4%)	6 (2.3%)	<0.01	8 (14.5%)	1 (0.6%)	<0.01	15 (57.7%)	5 (6.0%)	<0.01
Radical cystectomy	9 (11.1%)	43 (16.4%)	0.25	3 (5.5%)	7 (3.9%)	0.62	6 (23.1%)	36 (43.4%)	0.06
Comorbidity									
Diabetes mellitus	17 (21.0%)	44 (16.8%)	0.39	12 (21.8%)	32 (17.9%)	0.51	5 (19.2%)	12 (14.5%)	0.56
Heart Disease	16 (19.8%)	23 (8.8%)	0.01	12 (21.8%)	20 (11.2%)	0.04	4 (15.4%)	3 (3.6%)	0.03
Other malignancies	1 (1.2%)	11 (4.2%)	0.21	1 (1.8%)	5 (2.8%)	0.69	0 (0.0%)	6 (7.2%)	0.16
Chemotherapy in the past 1 year	4 (4.9%)	13 (5.0%)	0.99	0 (0.0%)	1 (0.6%)	0.58	4(15.4%)	12 (14.5%)	0.91
Radiotherapy	4 (4.9%)	7 (2.7%)	0.31	0 (0.0%)	2 (1.1%)	0.43	4 (15.4%)	5 (6.0%)	0.13

NMIBC, non-muscle-invasive bladder cancer; MIBC, muscle-invasive bladder cancer; SD, standard deviation; NTD, new Taiwan Dollar;
